# Nicotinic acetylcholine receptor agonist attenuates ILC2-dependent airway hyperreactivity

**DOI:** 10.1038/ncomms13202

**Published:** 2016-10-18

**Authors:** Lauriane Galle-Treger, Yuzo Suzuki, Nisheel Patel, Ishwarya Sankaranarayanan, Jennifer L. Aron, Hadi Maazi, Lin Chen, Omid Akbari

**Affiliations:** 1 Department of Molecular Microbiology and Immunology, Keck School of Medicine, University of Southern California, 1450 Biggy Street NRT 5509, Los Angeles, California 90033, USA; 2 Departments of Biological Science and Chemistry, University of Southern California, 1050 Childs Way RIH 201, Los Angeles, California 90089, USA

## Abstract

Allergic asthma is a complex and chronic inflammatory disorder that is associated with airway hyperreactivity (AHR) and driven by Th2 cytokine secretion. Type 2 innate lymphoid cells (ILC2s) produce large amounts of Th2 cytokines and contribute to the development of AHR. Here, we show that ILC2s express the α7-nicotinic acetylcholine receptor (α7nAChR), which is thought to have an anti-inflammatory role in several inflammatory diseases. We show that engagement of a specific agonist with α7nAChR on ILC2s reduces ILC2 effector function and represses ILC2-dependent AHR, while decreasing expression of ILC2 key transcription factor GATA-3 and critical inflammatory modulator NF-κB, and reducing phosphorylation of upstream kinase IKKα/β. Additionally, the specific α7nAChR agonist reduces cytokine production and AHR in a humanized ILC2 mouse model. Collectively, our data suggest that α7nAChR expressed by ILC2s is a potential therapeutic target for the treatment of ILC2-mediated asthma.

## Abstract (web summary)

Airway hyperreactivity is driven by type 2 cytokines produced by ILC2 and Th2 cells. Here the authors show that an α7-nicotinic receptor agonist (GTS-21) inhibits ILC2 responses and is therapeutic against *Alternaria*-induced airway hyperreactivity in a humanized mouse model.

## Graphical abstract

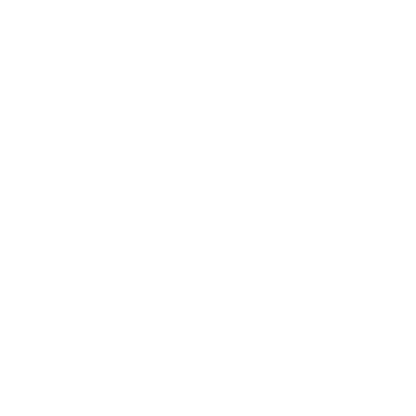

Asthma, which is a major worldwide health problem, is a chronic inflammatory disease of the airways with several phenotypes, comprised of both allergic and non-allergic asthma^1^,^2^. Allergic sensitization in which antigen-presenting cells (APCs) present allergens, followed by T-helper type 2 (Th2) cell skewing and eosinophilic inflammation, are essential for the development of allergic asthma. Obesity, ozone, viral infections, stress and air pollution are associated with non-allergic asthma, the pathogenesis of which involves the innate pathway rather than Th2 cell-mediated immunity^3^,^4^,^5^. Indeed, non-Th2 factors such as interferon-γ, IL-17 and neutrophils are often found in the lungs of patients with severe non-atopic asthma^1^,^2^. Moreover, these allergic and non-allergic components may be present in individual patients to various degrees, leading to a complex immune milieu and disease heterogeneity^1^,^2^.

Innate lymphoid cells (ILCs) are a non-B cell, non-T-cell lymphocyte population in mucosal and lymphoid tissues that are not antigen specific, but respond rapidly to environment factors to induce various types of cytokines^6^,^7^. Among the ILCs, group 2 ILCs (ILC2s) are directly activated by innate signals from myeloid and epithelial-derived cytokines and alarmins, such as IL-25, IL-33 and proteases, without requiring further differentiation. Following activation, ILC2s produce robust amounts of Th2 cytokines IL-5 and IL-13 to promote eosinophilic inflammation and airway hyperreactivity (AHR); thus, they play an essential role in the pathogenesis of asthma^6^,^7^. The suggestion that ILC2s are critical for innate immunity activation in asthma is logical as influenza infection^5^ and exposure to proteases and fungi^8^,^9^ induce AHR by activating innate lymphoid cells. In addition, ILC2s participate in shaping and regulating adaptive immune responses^10^. ILC2-produced IL-5 and IL-13 also contribute to asthma development by respectively recruiting eosinophils in airways and inducing goblet cell mucus production. ILC2s can also directly stimulate a Th2 response *in vitro*^11^ and facilitate an antigen-specific T-cell response during helminth infection^12^. This evidence suggests that ILC2s are deeply involved in the pathogenesis of asthma by modulating both innate and adaptive immune responses. Thus, regulating the function of ILC2s could be an ideal therapeutic strategy.

The α7 nicotinic acetylcholine receptor (nAChR), mediates rapid excitatory synaptic transmission and is shown to be a potential therapeutic target in neuropsychiatric^13^, neurodegenerative^14^ and inflammatory disease^15^,^16^. In pulmonary allergic inflammation, nicotine, an agonist for nAChR, attenuates Th2 cytokine, IgE and cysteinyl leukotriene levels, resulting in reduced allergic inflammation^17^,^18^. However, the specific cellular mechanisms by which nAChR activation regulates allergic inflammation are not clearly defined. Given the therapeutic potential of α7nAChR in neurological disorders and inflammatory diseases, a variety of α7-specific ligands have been developed by medicinal chemistry^19^,^20^ and structural approaches^21^,^22^,^23^. A number of these α7nAChR compounds showed promising efficacy and have advanced to clinical trials^19^. For the initial proof-of-concept studies presented here, we select a leading compound, GTS-21 (also known as DMXBA), that is functionally known to act as an agonist with partial selectivity toward α7nAChR^24^,^25^, and characterize its role in ILC2-dependent AHR. Utilizing this agonist, we demonstrate a critical role for α7nAChR in regulating ILC2-mediated AHR and airway inflammation in a preclinical model of allergic asthma. Moreover, we validate the specificity of the α7nAChR agonist using α7nAChR-deficient mice. As expected, the α7nAChR agonist was impaired in suppressing ILC2-dependent AHR in α7nAChR-deficient mice. Reconstitution studies with alymphoid mice suggest that while recipients of wild-type ILC2s responded to suppressive activity of the agonist, recipients of the α7nAChR-deficient ILC2s did not show any alteration in AHR or eosinophilia after agonist treatment. These observations establish that α7nAChR expression by ILC2s is crucial for the anti-inflammatory role of the agonist, and our reagent acts specifically through α7nAChR. Administration of the α7nAChR agonist inhibits activation of key transcriptional factors, such as GATA-3 and NF-κB. Furthermore, α7nAChR agonist abrogates phosphorylation of IKKα/β, a critical kinase upstream of the NF-κB signalling pathway. Using a translational approach, we also show that engagement of α7nAChR results in decreased cytokine production in human ILC2s and a reduction in AHR in a humanized ILC2 model. Our findings provide insight into the regulation of ILC2s that could ultimately be used to generate new therapeutic approaches for ILC2-dependent asthma.

## Results

### α7nAChR agonists and α7nAChR expression by ILC2s

**Figure 1:**
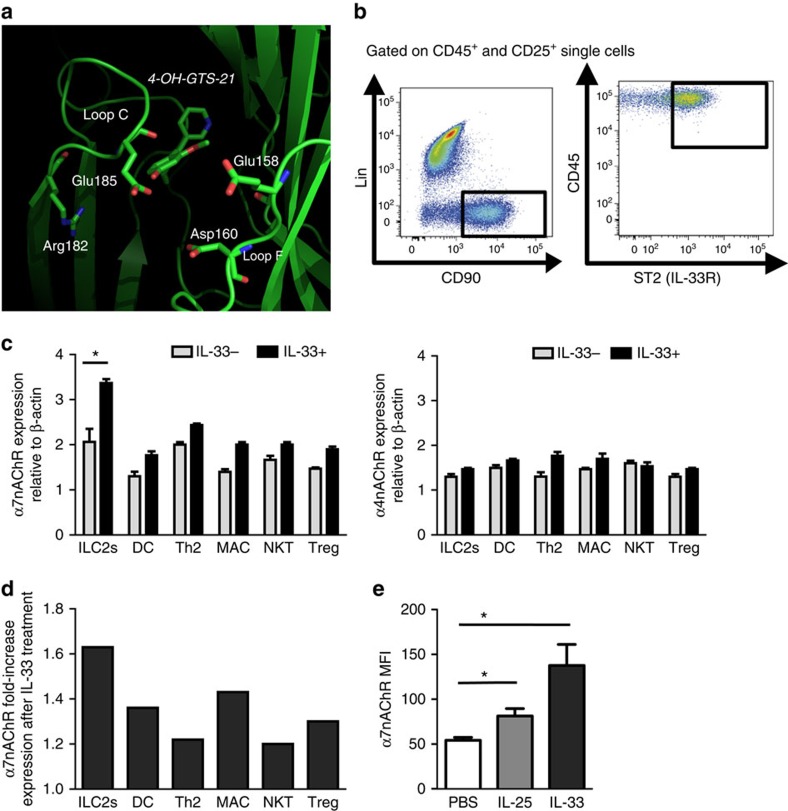
Structure of α7nAChR agonist and α7nAChR expression on ILC2s. (**a**) Structure of 4-OH-GTS-21 bound to the α7nAChR chimera. The receptor protein is shown in ribbon; the ligand and nearby protein residues are shown in stick model. The ligand, protein residues and key receptor loop (Loop C and Loop F) are labeled accordingly. (**b**) Flow cytometry analysis of lung ILC2s isolated from BALB/cByJ mice, as defined by a lack of lineage markers (CD3e, CD45R, Gr-1, CD11c, CD11b, Ter119, NK1.1, TCR-γδ and FcɛRI) and expression of CD90, CD45 and ST2. Dot plots show cells gated on CD45+ and CD25+ single cells. (**c**) Peripheral immune cells were sorted from naive BALB/cByJ mice and cultured with rm-IL-2 and rm-IL-7 in the presence or absence of rm-IL-33. The expressions of α7nAChR and α4nAChR were quantified at the mRNA levels by quantitative real-time PCR. (**d**) α7nAChR fold-change expression after IL-33 treatment in peripheral immune cells. (**e**) Expression of α7nAChR in isolated lung ILC2s from mice intranasally challenged for 3 consecutive days with PBS, IL-25 (5 μg), or IL-33 (0.5 μg). Data are representative of at least three independent experiments and are presented as means±s.e.m. (*n*=3; Student's *t*-test, *P<0.05).

Because of the potential therapeutic application in neurological disorders and inflammation, α7-selective ligands have been intensively studied. Li *et al*.^21^ solved the crystal structure of a receptor chimera constructed from the extracellular domain of α7nAChR and acetylcholine binding protein (AChBP), which represents the closest structural homologue of the native α7nAChR. We have also determined structures of α7nAChR bound by agonist and antagonist^21^,^26^, and performed structure-based screens of α7nAChR-specific compounds^22^. Our studies, together with those reported by others, have demonstrated that the crystal structure of the α7nAChR/AChBP chimera is a much better template for structure-based drug screening than AChBP^23^, which has been widely used in previous drug screening and design. These studies have identified a variety of potential ligands and allosteric modulators of α7nAChR, including many previously known α7-specific compounds. Of these, GTS-21 (DMXB-A) has been previously characterized as a neuronal nAChR ligand. GTS-21 binds to both the α4β2 and α7 subtypes^24^,^25^,^27^,^28^, but activates only the α7 subtype to a significant extent. In our structure-based drug screen, we selected 4-OH-GTS-21, the active form of GTS-21 *in vivo*, from the docking analyses. As shown in Fig. 1a, the overall structure is similar to that of 4-OH-GTS-21 bound to AChBP^29^, including the conformation and orientation of 4-OH-GTS-21 bound to the ligand pocket. On the other hand, our docking analyses reveal that a number of α7-specific residues located at loop C (Arg182 and Glu185) and loop F (Glu158 and Asp160), which are absent in AChBP, interact, or are poised to interact, with functional groups on 4-OH-GTS-21. These observations provide structural bases to guide further synthetic modification of GTS-21 to gain higher selectivity toward the α7 subtype and to modify the functional effects of similar ligands, thus expanding the therapeutic repertoire of agents based on the anabaseine scaffold.

The α7nAChR is present on macrophages^15^, T cells^30^,^31^ and B cells^32^, and activation of this receptor mediates anti-inflammatory effects. However, there are no studies investigating α7nAChR expression on ILC2s, despite their essential role in the development of asthma. We identified ILC2s as a Lin^-^, CD25^+^, CD45^+^, CD90.2^+^, and ST2^+^ population (Fig. 1b). As mentioned previously, ILC2s are activated by IL-33; however, they are not the only cells that express the IL-33 receptor. As GTS-21 binds to both α4 and α7 subtypes of nAChR, we further assessed mRNA levels of α7 and α4nAChR in various types of immune cells. Interestingly, α7nAChR expression was significantly higher in ILC2s as compared with other ST2^+^ peripheral immune cells (Fig. 1c). Importantly, only α7nAChR in ILC2s, but not α4nAChR, was significantly present and up-regulated by *in vitro* IL-33 treatment (Fig. 1c,d). To confirm this finding at the protein level and also to investigate the effect of IL-25 stimulation, we treated mice with intranasal recombinant mouse (rm)-IL-33, (rm)-IL-25, or PBS as a negative control, for three consecutive days. As shown in Fig. 1e, we found, for the first time, that α7nAChR is expressed on ILC2s. Importantly, α7nAChR expression on ILC2s was significantly upregulated in mice treated with IL-25 or IL-33, when compared with the PBS control group. α7nAChR expression was additionally confirmed by cytometry using fluorochrome-conjugated α-bungarotoxin, a nicotinic cholinergic blocker (Supplementary Fig. 1). Meanwhile, α7nAChR did not alter expression of CD25, CD127 and ST2, known as IL-2R, IL-7R and IL-33R, respectively, and which are essential for development of immune cells (Supplementary Fig. 2).

### α7nAChR agonist suppresses cytokine production in ILC2s

**Figure 2:**
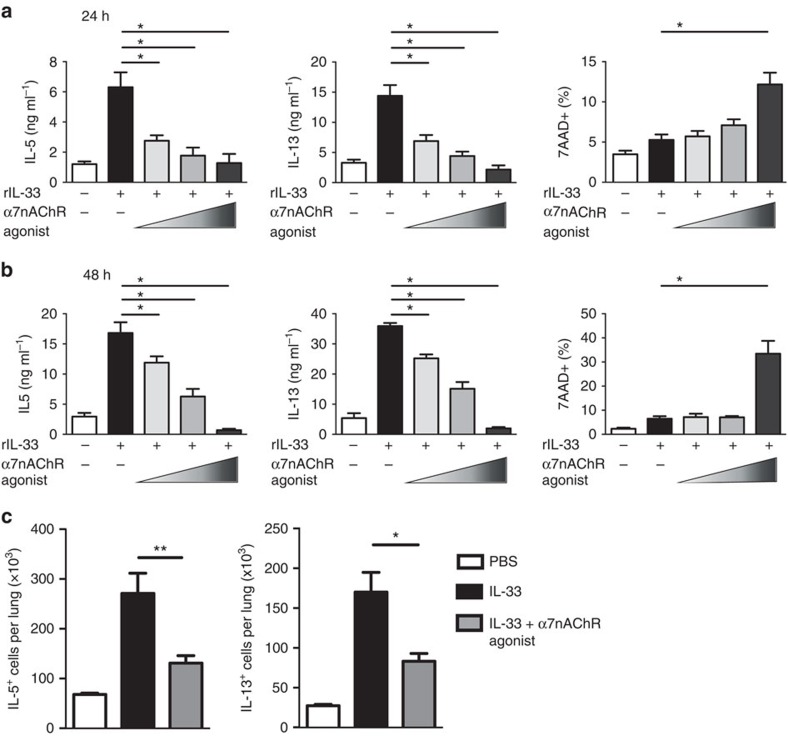
α7nAChR agonist suppresses IL-5 and IL-13 production from ILC2s *in vitro*. BALB/cByJ mice were intranasally challenged with recombinant mouse IL-33 on days 1–3. On day 4 lung ILC2s were isolated and re-stimulated with rIL-33, rIL-2 and rIL-7 with or without α7nAChR agonist (2.5, 10 and 50 μg ml^−1^) for 24 h (**a**) or 48 h (**b**). The levels of IL-5 and IL-13 were measured by ELISA. The viability of cultured ILC2s were assessed by staining with 7-AAD. (**c**) BALB/cByJ mice were intranasally challenged with recombinant mouse IL-33 on days 1-3, with or without α7nAChR agonist administration. On day 4 lung ILC2s were isolated and the number of IL-5^+^ and IL-13^+^ cells per 40 × 10^4^ ILC2s were quantified. Values are expressed as the mean±s.e.m. of five experiments (*n*=5; Student's *t*-test, *P<0.05; **P<0.01).

Nicotine is known as a major constituent of cigarette smoke, which causes impairment of lung function and exacerbation of asthma. Interestingly, nicotine administration attenuates production of Th2 cytokines and leukotrienes in preclinical models of asthma^17^. Nicotine is an agonist for a variety of pentameric nAChRs made up of different combinations of the sixteen nicotinic receptor subunits, thus it lacks specificity for α7nAChR^21^,^33^. Therefore, using GTS-21, an agonist specific for α7nAChR, we sought to determine whether engagement of α7nAChR would alter ILC2s' function. To address this question quantitatively, pulmonary ILC2s were isolated and cultured with increasing doses (2.5, 10 and 50 μg ml^−1^) of α7nAChR agonist in the presence of rm-IL-33, rm-IL-2 and rm-IL-7. Our results show dose-dependent anti-inflammatory effects of α7nAChR agonist on ILC2s (Fig. 2). These effects were independent of cell viability except at the highest tested dose of the agonist (Fig. 2a,b, right panels). To verify that the actions of the agonist were not due to a reduction in the number of ILC2s, we administered IL-33 to mice *in vivo* with or without agonist treatment, and then quantified the number of IL-5^+^ and IL-13^+^ ILC2s within a determined number of ILC2s (Fig. 2c). Similarly to IL-33, IL-25 increased IL-5 and IL-13 secretion in ILC2s *in vitro*, though this effect was less robust. IL-25-induced cytokine secretion was also susceptible to suppression by the agonist (Supplementary Fig. 3).

### α7nAChR agonist treatment ameliorates ILC2-induced AHR

**Figure 3:**
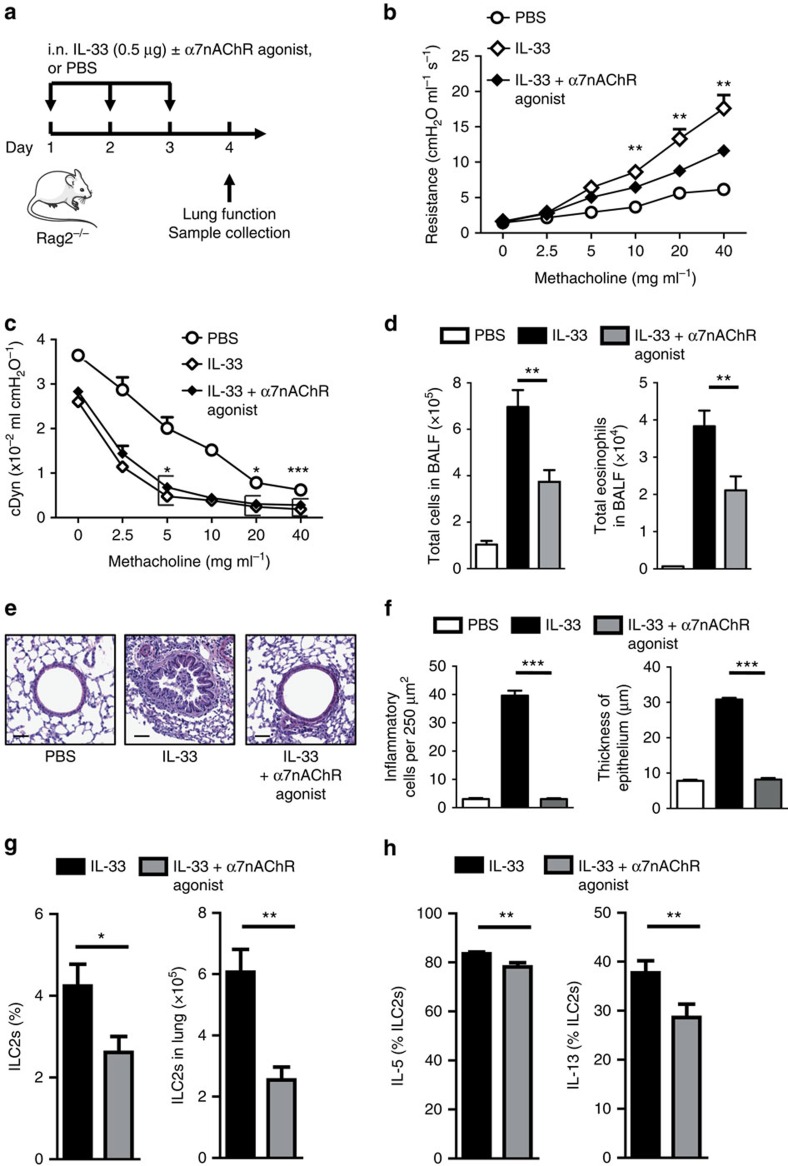
α7nAChR agonist treatment ameliorates ILC2s-mediated AHR. (**a**) Rag2-deficient mice were intranasally challenged with recombinant mouse IL-33 or PBS and also received α7nAChR agonist (125 μg) or PBS on days 1–3 according to **a**. Measurement of lung function and analyses of bronchoalveolar lavage fluid (BALF) and lung histology followed on day 4. (**b**,**c**) Lung resistance and dynamic compliance. (**d**) Total number of cells and eosinophils in BALF. (**e**) Hematoxylin and eosin-stained lung sections ( × 200). Scale bars, 50 μm. (**f**) Quantification of lung histopathology shown as number of inflammatory cells per 250 μm^2^ and thickness of airway epithelium (μm). (**g**) Total number and frequency of lung ILC2s. (**h**) Percentage of IL-5^+^ and IL-13^+^ lung ILC2s determined by flow cytometry. Data are representative of at least four independent experiments and are presented as means±s.e.m. (*n*=8; Student's *t*-test, *P<0.05; **P<0.01; ***P<0.001). Mouse outline image provided with permission from Servier Medical Art.

As reported previously^6^,^7^,^8^,^9^,^11^,^12^,^34^,^35^, the IL-5 and IL-13 cytokines produced by activated ILC2s are essential for eosinophilic inflammation and AHR development. We investigated whether the attenuated ILC2 function by α7nAChR stimulation *in vitro* could result in inhibition of ILC2-mediated AHR and allergic inflammation. Rag2 deficient mice (devoid of T and B cells) were given intranasal (i.n.) rm-IL-33, with or without α7nAChR agonist for three consecutive days (Fig. 3a). As IL-33 administration specifically induces ILC2s, thereby causing AHR, with this model we can readily explore the effect of an α7nAChR agonist in ILC2-mediated AHR. One day after the last challenge, lung function was evaluated by direct measurements of lung resistance (*R*_L_) and dynamic compliance (*C*_dyn_), as described in Methods section. We found that stimulating α7nAChR significantly reduced levels of *R*_L_ and of *C*_dyn_ in response to IL-33, as compared with PBS (Fig. 3b,c). Bronchoalveolar lavage fluid (BALF) analyses also showed decreased eosinophilic infiltration, as well as total cell counts, in Rag2^*−/−*^ mice treated with the α7nAChR agonist (Fig. 3d). Histological analyses revealed that α7nAChR stimulation prevented airway wall thickness and infiltrated cells (Fig. 3e,f). As shown in Fig. 3g, treatment with GTS-21 also significantly suppressed the frequency and absolute number of lung ILC2s. Furthermore, the intracellular cytokine assay also revealed significantly decreased levels of IL-5 and IL-13 producing lung ILC2s in α7nAChR agonist treated mice compared with untreated mice (Fig. 3h). These findings concur with the reduction of AHR and eosinophil counts in BALF (Fig. 3b–d). Thus, α7nAChR agonist represses IL-5 and IL-13 production, eosinophil recruitment, and ILC2-dependent AHR.

### α7nAChR on ILC2s is critical for GTS-21 action *in vivo*

**Figure 4:**
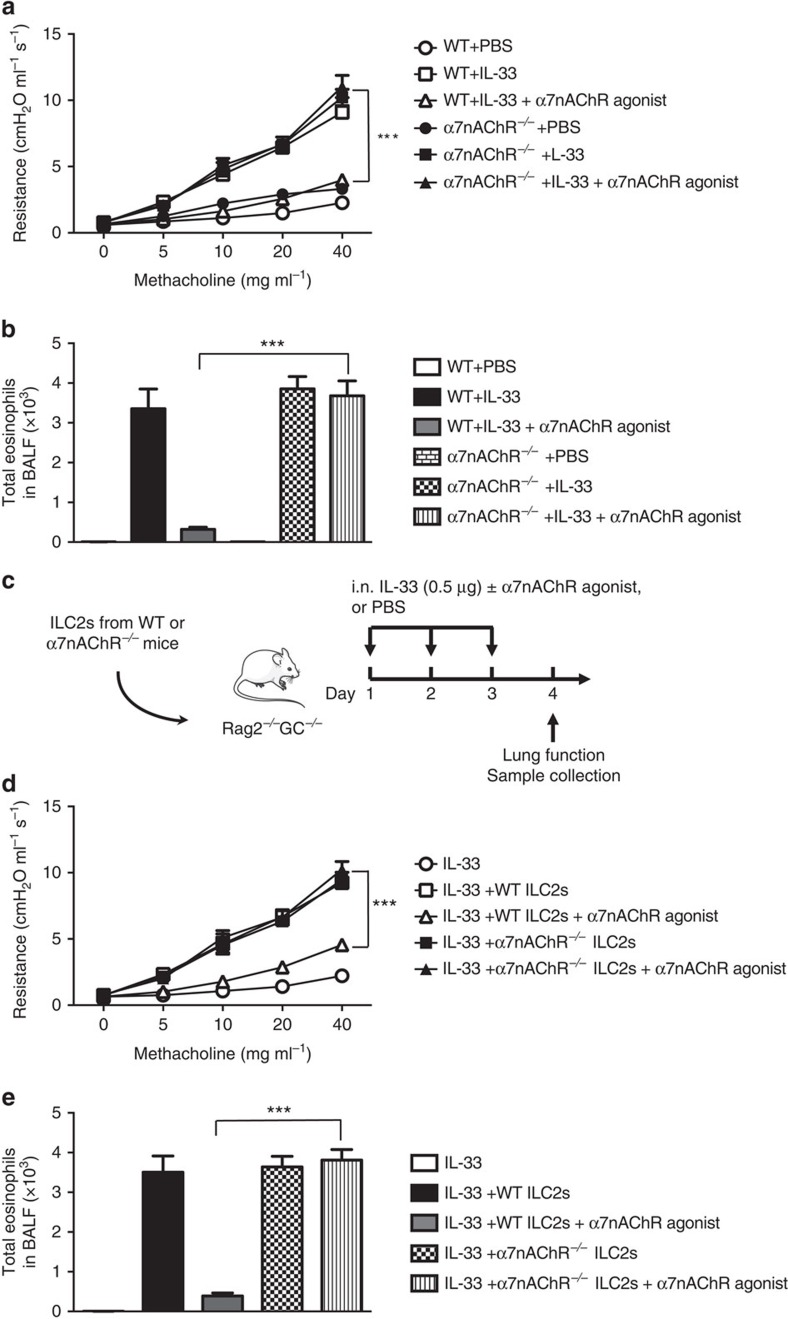
α7nAChR expression is critical for GTS-21 action on ILC2s *in vivo*. BALB/cByJ and α7nAChR^*−/−*^ mice were intranasally challenged with rm-IL-33 or PBS, and also given α7nAChR agonist (125 μg) or PBS on days 1–3. Measurement of lung function and analysis of BAL followed on day 4. (**a**) Lung resistance. (**b**) Total number of eosinophils in BALF. (**c**) Rag^*−/−*^ GC^*−/−*^ mice were received ILC2s from either WT or α7nAChR^*−/−*^ mice. After the adoptive transfer, mice were intranasally challenged with rm-IL-33 or PBS; they also received α7nAChR agonist (125 μg) or PBS on days 1–3. Measurement of lung function and analysis of BAL followed on day 4, according to **c**. (**d**) Lung resistance. (**e**) Total number of eosinophils in BALF. Data are representative of at least three independent experiments and are presented as means±s.e.m. (*n*=4; Student's *t*-test, ***P<0.001, WT IL-33+α7nAChR agonist vs. α7nAChR^*−/−*^ IL-33+α7nAChR agonist). Mouse outline image provided with permission from Servier Medical Art.

To assess whether the previously observed effects of GTS-21 are due to its actions on the α7nAChR, we challenged WT and α7nAChR-deficient mice with or without rm-IL-33, and with or without α7nAChR agonist, for three consecutive days (Fig. 4a,b). As expected, in the WT mice, the agonist repressed IL-33-induced AHR and eosinophilic infiltration. However, in the absence of α7nAChR, the agonist affected neither AHR nor eosinophilia. Taken together, these results indicate that engagement of α7nAChR ameliorates ILC2-mediated AHR and allergic inflammation.

Given that a variety of cells participate in allergic inflammation, we wanted to investigate the effect of the agonist specifically on ILC2s. Rag2^*−/−*^ GC^*−/−*^ mice lack not only B and T cells, but NK cells and ILC2s as well. Using methods previously described by our laboratory^36^, we adoptively transferred WT or α7nAChR-deficient ILC2s into these Rag2^*−/−*^ GC^*−/−*^ mice, and then treated them with IL-33, with or without α7nAChR agonist. As expected, we observed a decrease of AHR and of eosinophil recruitment in mice injected with WT ILC2s in response to α7nAChR treatment (Fig. 4c-e). However, in the absence of α7nAChR expression on ILC2s, the agonist affected neither AHR nor eosinophilia. Taken together, these results indicate that engagement of α7nAChR ameliorates ILC2-mediated AHR and allergic inflammation. These results, which are consistent with those seen in WT and α7nAChR-deficient mice, demonstrate that the effects we observed are due to ILC2s as opposed to other cells, such as structural cells, which express α7nAChR.

### α7nAChR agonist attenuates GATA3 and NF-κB expression

**Figure 5:**
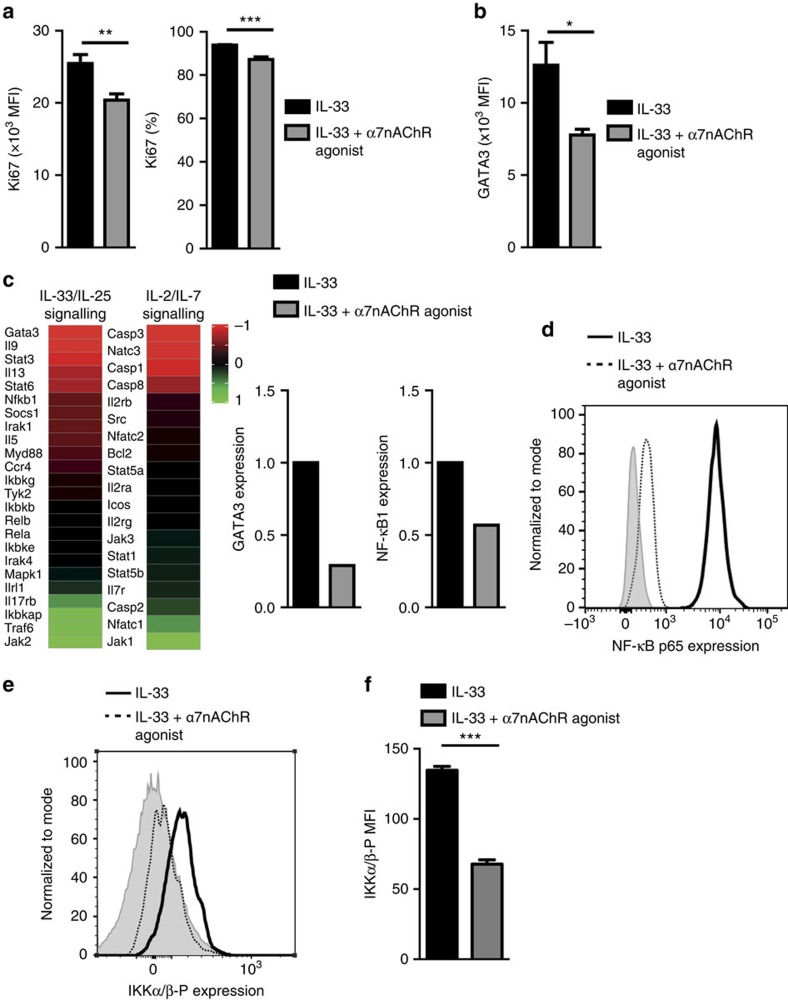
Cholinergic signal attenuates ILC2 proliferation and GATA3 expression. (**a**) Mean fluorescence intensity of Ki67 and percentage of Ki67+ cells in isolated lung ILC2s, stimulated with IL-33 in the absence and presence of α7nAChR. (**b**) Mean fluorescence intensity of GATA3 in isolated lung ILC2s, stimulated with IL-33 in the absence and presence of α7nAChR. One representative experiment of two is shown (*n*=5; Student's *t*-test, *P<0.05; **P<0.01; ***P<0.001). (**c**) Heat plot demonstration of modulation of depicted genes in lung ILC2s treated with α7nAChR agonist. FACS-purified ILC2s from WT mice challenged with rm-IL-33 with or without α7nAChR agonist as described in Fig. 3a were quantified by NanoString nCounter technology. Data range from −1 to +1 for the most reduced and most increased gene expression, respectively. (**d**) Expression of NF-κB p65 in isolated lung ILC2s from mice challenged with IL-33 with (dotted line) or without (thick line) α7nAChR agonist. The level of isotype-matched stain control is shown as a grey-filled histogram. (**e**) Expression of phosphorylated IKKα/β (Ser176/180) in isolated lung ILC2s stimulated with IL-33, with (dotted line) or without (thick line) α7nAChR agonist for 24 h. The level of isotype-matched stain control is shown as a grey-filled histogram. (**f**) Mean fluorescence intensity of phosphorylated IKKα/β with or without α7nAChR agonist.

To characterize the mechanism enabling α7nAChR agonist to attenuate ILC2-mediated AHR, we first investigated whether engagement of α7nAChR affects the development or maintenance of pulmonary ILC2s. However, we observed a significantly decreased proliferation rate of pulmonary ILC2s upon α7nAChR agonist treatment by evaluating Ki-67 levels (Fig. 5a). We also evaluated the expression of the transcription factor GATA binding protein-3 (GATA-3), which is essential for the development and maintenance of ILC2s^37^,^38^,^39^. We found that α7nAChR agonist treatment significantly inhibits GATA-3 transcription in ILC2s (Fig. 5b). This decrease in GATA-3 expression could be involved in the repressed proliferation and function of ILC2s in response to α7nAChR agonist.

Next, to investigate the molecular mechanism of α7nAChR agonist-mediated anti-inflammatory effects, we analysed the gene expression profile of ILC2s with or without α7nAChR agonist treatment by NanoString technology as described in Methods section. Evaluated genes were categorized and displayed in two panels; (1) genes mainly involved in the IL-33/IL-25 signalling pathway, and (2) genes associated with IL-2/IL-7 signalling pathway (Fig. 5c). As demonstrated in the IL-33/IL-25 signalling pathway, α7nAChR-mediated cholinergic activity inhibited expression of GATA-3 as well as STAT-6, NF-κB, IL-5, IL-9 and IL-13. Meanwhile, STAT-5a, STAT-5b and IL-2 receptors in the IL-2/IL-7 signalling panel were relatively unaffected. We also demonstrated that α7nAChR agonist did not activate factors associated with apoptosis, such as Casp3, Casp8, and Bcl-2. We further confirmed by flow cytometry that α7nAChR agonist reduced NF-κB p65 expression in ILC2s (Fig. 5d). To better establish the underlying mechanisms, we explored the signalling pathway upstream of NF-κB by measuring the activated form of IKKα/β, which is phosphorylated on serine residues 176 and 180. Consistent with our previous results shown in Fig. 5d, α7nAChR agonist reduced phosphorylated IKKα/β expression in ILC2s (Fig. 5e,f). NF-κB and STAT-6 are both critical for GATA-3 transcription. The inhibition of GATA-3 expression results in a down-regulation of IL-5, IL-9 and IL-13 secretion from ILC2s. Strikingly, these results demonstrate that α7nAChR stimulation likely reduces maintenance and development of ILC2s by inhibiting GATA-3 transcription, resulting in marked anti-inflammatory effects in the development of ILC2-mediated AHR and allergic inflammation.

### α7nAChR agonist treatment attenuates allergen-induced AHR

**Figure 6:**
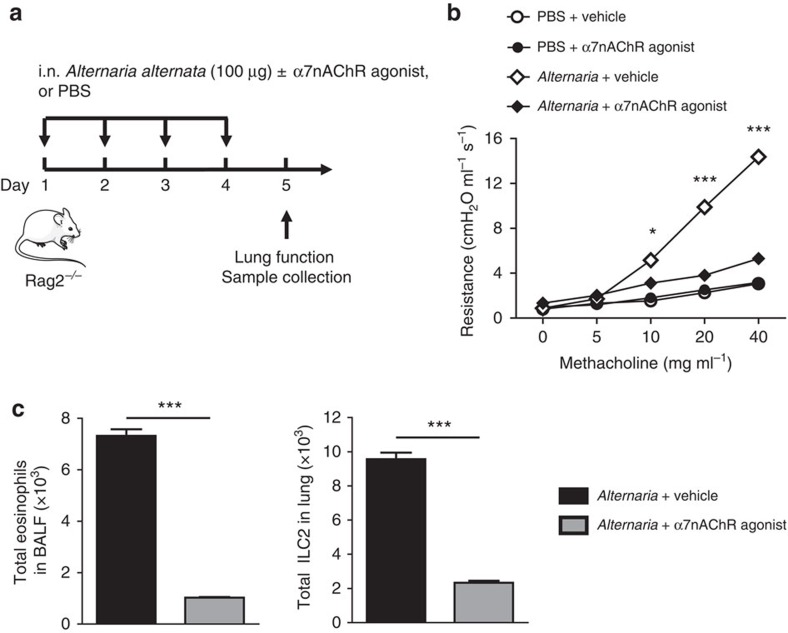
α7nAChR agonist inhibits *Alternaria*-induced AHR. (**a**) Rag2-deficient mice intranasally received an extract of *Alternaria alternata* with or without α7nAChR agonist on days 1-4 according to **a**. Measurement of lung function and BALF analysis followed on day 5. (**b**) Lung resistance. (**c**) Total number of eosinophils in BALF. Total number of lung ILC2s. Data are representative of at least four independent experiments and are presented as means±s.e.m. (*n*=4; Student's *t*-test, *P<0.05; ***P<0.001). Mouse outline image provided with permission from Servier Medical Art.

It has been previously reported that *Alternaria alternata* can induce AHR^40^. We further explored the anti-inflammatory effects of α7nAChR agonist in ILC2-mediated AHR with this clinically relevant allergen. Rag2^*−/−*^ mice were i.n. administered *A. alternata* extract with or without α7nAChR agonist for 4 consecutive days (Fig. 6a). As expected, α7nAChR agonist treated mice did not develop AHR (Fig. 6b). Accordingly, the number of eosinophils in BALF and lung ILC2s were increased in *Alternaria*-treated mice. Meanwhile, α7nAChR agonist treatment abolished eosinophils and ILC2s in the lung (Fig. 6c). These results suggest α7nAChR agonist treatment can attenuate ILC2-mediated AHR in response to other allergens besides IL-33.

### α7nAChR agonist prevents AHR in humanized ILC2 mice

**Figure 7:**
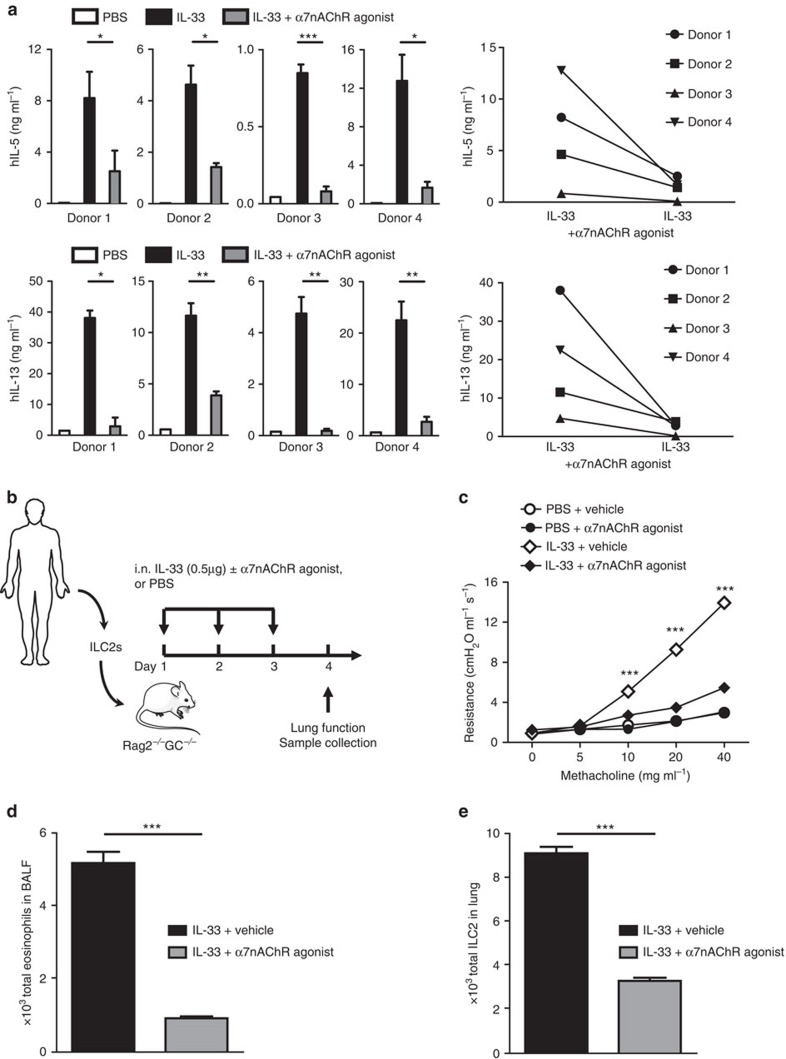
α7nAChR agonist prevents human ILC2s mediated AHR and allergic. (**a**) Human ILC2s were purified from PBMCs via FACS and cultured (10^4^ cells per ml) in the presence of recombinant human IL-33 (20 ng ml^−1^), IL-2 (10 ng ml^−1^) and IL-7 (20 ng ml^−1^), with or without α7nAChR agonist for 24 h. In the right panel, data are presented as means of four individual donors. (**b**) Human peripheral blood mononuclear cells were isolated via FACS and cultured in the presence or absence of rh-IL-2 (10 ng ml^−1^) and rh-IL-7 (20 ng ml^−1^) for 48 h, then adoptively transferred into Rag2^*−/−*^ GC^*−/−*^ mice that were intranasally challenged with rm-IL-33 or PBS, with or without α7nAChR agonist (125 μg) on days 1–3. Measurement of lung function and analysis of BALF followed on day 4. (**c**) Lung resistance. (**d**) Total number of eosinophils in BALF. (**e**) Total number of lung ILC2s. Data are representative of at least two independent experiments and are presented as means±s.e.m. (*n*=4; Student's *t*-test, *P<0.05; **P<0.01; ***P<0.001). Mouse and human outline image are provided with permission from Servier Medical Art.

To assess the effects of α7nAChR agonist in human ILC2s, peripheral blood mononuclear cells (PBMCs) were obtained from healthy donors and ILC2s were sorted on the basis of expression of CD45, CRTH2, CD127 and CD161, and the lack of human lineage markers (CD3, CD14, CD16, CD19, CD20, CD56, CD235a, CD1a and CD123). Sorted ILC2s were cultured with α7nAChR agonist (10 μg ml^−1^) in the presence of recombinant human (rh)-IL-2 (10 ng ml^−1^), rh-IL-7 (20 ng ml^−1^) and rh-IL-33 (20 ng ml^−1^). As observed in our previous experiments (Fig. 2a,b), α7nAChR agonist significantly suppressed human ILC2 activation, by decreasing both IL-5 and IL-13 production (Fig. 7a).

Finally, to confirm the efficacy of α7nAChR agonist treatment in human ILC2-mediated AHR, sorted human ILC2s (hILC2s) were cultured in the presence of rh-IL-2 (20 ng ml^−1^) and rh-IL-7 (20 ng ml^−1^) for 48 h. Subsequently, 2.5 × 10^5^ hILC2s were adoptively transferred to Rag2 GC double knockout mice, which lack T, B, NK cells and ILC2s. These humanized mice were given i.n. rh-IL33 (1 μg per mice) with or without α7nAChR agonists (125 μg ml^−1^) for three consecutive days, and then AHR was evaluated (Fig. 7b). Consistent with the murine ILC2 experiments, treatment with α7nAChR agonist dampened ILC2-mediated AHR (Fig. 7c) and allergic inflammation (Fig. 7d,e) in humanized mice. Thus, α7nAChR mediates an anti-inflammatory signal in both murine and human ILC2s, suggesting α7nAChR activation with an agonist as a novel potential therapeutic strategy for regulating ILC2-mediated lung inflammatory diseases.

## Discussion

In the present study, using a leading compound with good selectivity and partial agonist activity towards α7nAChR, we examined the involvement of α7nAChR in the pathogenesis of asthma to evaluate its potential as a therapeutic target. We demonstrated, for the first time, that α7nAChR is expressed on ILC2s and upregulated after engagement of the ST2 receptor by IL-33. By inhibiting GATA-3 transcription in ILC2, administration of α7nAChR agonist significantly attenuated the development and function of ILC2s, abolishing both AHR and allergic inflammation. Importantly, we showed this agonist also ameliorated human ILC2-mediated AHR, indicating its potential as a novel therapeutic molecule for asthma patients.

α7nAChR is a neuronal subtype of nAChR composed of a homopentamer of α7 subunits that mediates pre- and post-synaptic excitation. It is known that α7nAChR is located not only in the brain but also in the periphery, including immune cells such as effector T cells^30^,^31^, B cells^32^, Tregs^41^ and macrophages^15^. Using an improved template based on crystal structures, we performed a virtual screen for α7nAChR ligands and selected a leading compound, GTS-21, that has been previously characterized as a functional agonist of α7nAChR. Although GTS-21 had previously been shown to have anti-inflammatory effects^42^,^43^, its role in ILC2-dependent AHR has not been examined to our knowledge. In line with previous studies, we examined a newly identified lymphocyte population of ILC2s and showed that ILC2s expressed α7nAChR. Importantly, activated ILC2s upregulated α7nAChR expression more so than other immune cells, indicating that asthma patients having activated ILC2s might benefit greatly from a nicotinic agonist compound such as GTS-21.

Nicotine is just one of the over 4,000 chemical constituents in tobacco smoke. Exposure to cigarette smoking causes impaired lung function and increases the risk of developing asthma^44^,^45^. In asthma, patients who smoke have more symptoms and exacerbations than non-smokers^46^. They also have increased risks of hospitalization and mortality^47^. On the other hand, epidemiological studies have indicated inverse correlations between smoking and incidence of allergic diseases. It is well known that the incidence of hypersensitive pneumonitides such as farmer's lung and bird fancier's lung is lower in the current smoker population than that in non-smokers^48^,^49^,^50^,^51^. Furthermore, other clinical studies have observed that asthma incidence could be higher in former smoker populations compared with active smokers^44^,^52^. In that longitudinal study, the observed increased asthma risks in former smokers were explained by the fact that, in some cases, asthma was self-reported or the supposition that people tend to quit smoking in response to respiratory symptoms. A cohort study also showed that development of allergic sensitization is negatively associated with sustained smoking^53^. In accordance with these clinical findings, nicotine, a major constituent of cigarette smoke and a ligand for nAChR, has shown anti-inflammatory effects in various diseases^31^,^54^,^55^. In asthma, nicotine attenuated HDM-induced allergic lung inflammation together with suppression of Th2 cytokines, although the underlying cellular mechanism remains unknown and AHR was not suppressed^17^. Moreover Kearley *et al*.^56^ recently demonstrated that cigarette smoke has suppressive effects on IL-5 and IL-13 production by ILC2s. In preclinical models of asthma, we clearly demonstrated engagement of α7nAChR opposes the development of AHR and allergic inflammation through an ILC2-mediated mechanism. The differences in outcome likely resulted from the experimental design; our group administered a specific compound for α7nAChR together with rm-IL-33 or *A. alternata* to mice, whilst Sopori *et al*. examined nicotine itself and HDM-induced AHR^21^,^33^. To establish that our previous observations were dependent of α7nAChR expression on ILC2s, we demonstrated that the α7nAChR agonist had no effect on IL-33-challenged mice that were deficient for α7nAChR on ILC2s. Collectively, our data can explain the cellular mechanisms behind the clinical anti-inflammatory observations.

We demonstrated that α7nAChR activation altered ILC2 function in response to both exogenous IL-33 and *A. alternata.* Administration of either IL-33 and IL-25 activates ILC2s to induce AHR independently of the adaptive immune system^5^,^6^,^57^, and IL-33 was reported to be more potent than IL-25 in this regard^57^. *A. alternata* exposure also activates ILC2s and causes steroid-resistant AHR associated with elevated IL-33 *in vivo*^40^. ILC2s rapidly produce huge amounts of IL5 and IL-13 in response to stimuli. A recent study showed that ILC2-derived IL-13 is capable of inducing differentiation among Th2 cells^35^. Based on these reports, regulating ILC2s could potentially control allergic inflammation arising from not only the innate, but also adaptive, immune pathway. By targeting ILC2 function, α7nAChR agonist treatments could prove remarkably therapeutic for various allergic diseases.

To explore the mechanisms underlying the anti-inflammatory effects of the α7nAChR agonist, we initially demonstrated the engagement of α7nAChR caused reduced number of ILC2s in the lung, suggesting involvement of the cholinergic signal in cell fate and maintenance. We next found that α7nAChR agonist significantly suppressed Ki67 expression, a cellular marker for proliferation in ILC2s. In contrast, expression of anti-apoptotic factor Bcl-2 on ILC2s was unaffected by α7nAChR stimulation. In accordance with these *in vivo* data, viability of ILC2s in culture and results from the NanoString assay were comparable. These results suggest that cholinergic signal transmission regulates proliferation, but not cell death, in ILC2s. Our findings are consistent with the cholinergic underpinnings of anti-inflammatory mechanisms in other diseases of inflammation, such as autoimmune arthritis and experimental autoimmune encephalomyelitis^31^,^54^,^55^.

On one hand, Dowling *et al*.^58^ also described that nicotine could inhibit the NF-κB pathway in an α7nAChR-dependent manner. We demonstrated by cytometry that in response to the agonist, the expression of the activated NF-κB p65 subunit was reduced. Similarly we also observed a decrease in the expression at the mRNA level of NF-κB1. However, with the Nanostring Technology we also noticed that RelA expression was unaffected. This discrepancy could be explained by the fact that NF-κB is mainly regulated at the post-translational level by phosphorylation. Phosphorylation of the NF-κB p65 subunit on certain residues plays a key role in regulating NF-κB activation and function. We also validate our results by assessing IKKα/β, upstream of NF-κB and observed a significant reduction in IKKα/β after agonist treatment.

On the other hand, in cancer immunity, nicotine administration enhances tumour growth by promoting cell proliferation and suppressing apoptosis^59^,^60^,^61^,^62^. Strikingly, these results suggest that cholinergic signal is involved in the pathogenesis of various diseases, with a distinct role in each disease.

In addition to attenuated proliferation, α7nAChR agonist also inhibited GATA-3 expression in ILC2s. GATA-3, a double zinc-finger transcription factor, is required for the development of Th2 cells and important for the production of IL-5 and IL-13 (refs 63, 64, 65). Recently, GATA-3 was also reported to be essential for differentiation and maintenance of ILC2s, and their production of IL-5 and IL-13 (refs 37, 38, 39). Moreover, higher expression of GATA-3 is associated with an increase in ILC2-derived IL-13 (ref. 66). Collectively, these results indicate an underlying mechanism for the anti-inflammatory role of α7nAChR agonist in asthma: modulating GATA-3 expression and proliferation in ILC2s, which subsequently attenuates Th2 cytokine production from ILC2s, preventing the development of AHR and allergic inflammation.

We further investigated whether this cholinergic anti-inflammatory effect was relevant in a humanized mice model, in which human ILC2s were adoptively transferred to Rag^*−/−*^ IL2rg^*−/−*^ mice and administered IL-33 to induce AHR. This unique system allows one to directly evaluate human ILC2-mediated AHR^36^. In accordance with the effects seen in murine ILC2s, α7nAChR agonist significantly suppressed human ILC2 function and dampened human ILC2-mediated AHR. Taken in their entirety, our results suggest that enhancing cholinergic signal might be an effective therapeutic strategy for patients with ILC2-mediated asthma.

In conclusion, this is the first report to reveal that the engagement of α7nAChR on ILC2s suppresses AHR. Therefore, our results suggest a protective role of cholinergic signalling in the pathogenesis of asthma, and present α7nAChR agonists as novel therapeutic candidates for controlling ILC2-mediated inflammatory lung diseases.

## Methods

### Mice

Female BALB/cByJ, RAG2 deficient (C.B6(Cg)-Rag2^tm1.1Cgn^/J) mice, RAG2 GC deficient (C; 129S4-Rag2^tm1.1Flv^ IL2rg ^tm1.1Flv^ /J) mice and α7nAChR deficient (B6.129S7-Chrna7^tm1Bay^/J) (6–8 weeks old) were purchased from Jackson Laboratory (Bar Harbor, ME). Rag2-deficient and Rag2 GC-deficient mice were bred in our facility at the Keck School of Medicine, University of Southern California (USC). Animal studies were approved by the USC Institutional Animal Care and Use Committee and conducted in accordance with the USC Department of Animal Resources' guidelines. All human studies were approved by USC institutional review board and conducted according to the principles of the Declaration of Helsinki. Participants gave written informed consent to before their inclusion in the study, and were identified by number.

### Crystallization and structure determination of agonist

The structure of the agonist 4-OH-GTS-21 was docked into the ligand site of the crystal structure of the α7nAChR chimera^21^ using our previously published computation docking procedures^22^. The figure is made by PyMOL (The PyMOL Molecular Graphics System, v 1.8, Schrodinger, LLC).

### Measurement of airway hyperreactivity

Mice were i.n. administered carrier-free recombinant mouse IL-33 (BioLegend, San Diego, CA, 0.5 μg per mouse in 50 μl) with or without 125 μl of α7nAChR agonist (kindly provided by Lin Chen) on 3 consecutive days. Mice were i.n. administered carrier-free recombinant mouse IL-25 (BioLegend, San Diego, CA, 5 μg per mouse in 50 μl) with or without 125 μl of α7nAChR agonist on three consecutive days. For *Alternaria alternata* experiments, mice were i.n. administered *A. alternata* (Greer Labs, Lenoir, NC, 100 μg per mouse in 50 μl) with or without 125 μg α7nAChR agonist for 4 consecutive days. One day after the last challenge, mice were anesthetized using i.p. injection of ketamine (10 mg ml^−1^) and xylazine (1 mg ml^−1^). Measurements of airway resistance and dynamic compliance were conducted with the Fine Pointe RC System (Buxco Research Systems, Wilmington, NC), in which mice were mechanically ventilated using a modified version as previously described^36^,^67^. Mice were sequentially challenged with aerosolized PBS (baseline), followed by increasing doses of methacholine. Maximum lung resistance (*R*_L_) and minimum compliance (*C*_dyn_) values were recorded during a 3-min period after each methacholine challenge.

### Collection of BAL cells and lung histology

After measurements of AHR, the trachea was canulated and the lungs lavaged three times with 1 ml ice cold PBS to collect BALF cells as previously described^68^. BALF cells were stained with allophycocyanin (APC)-labeled anti-Ly-6G/Ly-6C (clone RB6-8C5, BioLegend, San Diego, CA), Alexa Fluor-labeled anti-CD19 (clone 6D5, BioLegend), phycoerythrin (PE)-labeled anti-Singlec-F (clone E50-2440, BD Pharmingen, San Diego, CA), and PE-Cy (PE-Cy7) labeled anti-CD45 (clone 30-F11, BioLegend), peridinin-chlorophyll-protein complex-Cy5.5 (PerCP-Cy5.5) labeled anti-CD3e (clone145-2C11, eBioscience, San Diego), and eFluor-450 labeled anti-CD11b (clone M1/70, eBioscience), and APC-Cy7 labeled anti-CD11c (clone N418, BioLegend). Transcardinal perfusion of the lungs with cold PBS was then performed to remove red blood cells and the lungs fixed and harvested for histology with 4% paraformaldehyde in PBS. After fixation, the lungs were embedded in paraffin, cut into 4 μm sections, and stained with hematoxylin and eosin (H&E).

### Flow cytometry

Biotinylated anti-mouse lineage (CD3e (145-2C11), CD45R (RA3-3B2), Gr-1 (RB6-8C5), CD11c (N418), CD11b (M1/70), Ter119 (TER-119), NK1.1 (PK136), TCR-β (H57-597), TCR-γδ (GL3), and FcɛRIα (MAR-1), Streptavidin-FITC, FITC anti-mouse CD3 (145-2C11), BV510 anti-mouse CD4 (RM4-5), APC-Cy7 anti-mouse CD25 (PC61), PE anti-mouse CD25 (PC61), BV421 anti-mouse CD25 (PC61), BV510 anti-mouse CD90.2 (53-2.1), APC anti-mouse CD127 (A7R34), PE-Cy7 anti-mouse CD127 (A7R34), FITC anti-mouse CD45 (30F-11), PE/Cy7 anti-mouse CD45 (30-F11), PE anti-mouse IL-5 (TRFK5), BV421 anti-mouse GATA3 (16E10A23), 7-AAD Viability staining solution, purchased from BioLegend (San Diego, CA). PE anti-mouse ST2 (IL-33R, RMST2-2), PerCP-eFluor710 anti-Mouse ST2 (RMST2-2), Streptavidin APC-eFluor780, Alexa Fluor647 labeled anti-IL-4 (clone 11B11), eFluor 450 anti-mouse CD45 (30F-11), eFluor450 anti-CD44 (clone IM7), eFluor-660 anti-mouse Ki-67 (SollA15), PE/Cy7 anti-mouse IL-13 (eBio13A), anti-mouse BCL-2 (10C4), were purchased from eBioscience. Alexa Fluor 488 anti-mouse p65-NF-κB (532301) was purchased from R&D Systems (dilution 1:50). Alexa Fluor 647 conjugated α-bungarotoxin (Invitrogen, San Diego, CA). PE anti-mouse Phospho-IKKα/β (Ser 176/180) (16A6) was purchased from Cell Signaling Technology (dilution 1:50). PE anti-rat α7nAChR (319) was purchased from Santa Cruz Biotechnology (dilution 1:50). This antibody has been used to identify this receptor previously in several studies^69^. Lineage marker antibodies were used at the dilution of 1:400, unless mentioned otherwise other antibodies were used at the dilution of 1:200.

BD Cytofix Fixation Buffer and BD Phosflow Perm Buffer III were purchased from BD Biosciences (San Jose, CA). Flow cytometry was carried out on the FACSCanto II and FACSARIA III (BD Biosciences) and the data were analysed with FlowJo version 8.6 software (TreeStar, Ashland, Oregon).

### Identification of mouse ILC2s

Lung ILC2s were defined as lack of classical lineage markers (CD3e, CD45R, Gr-1, CD11c, CD11b, Ter119, NK1.1, TCR-β, TCR-γδ and FcɛRIα) and CD25^+^, CD45^+^, CD90.2^+^, and ST2^+^ populations.

### Intracellular staining

Intracellular staining was performed using BD Cytofix/Cytoperm kit (BD Bioscience, San Jose, CA) according to the manufacturer's instructions. For analysis of GATA3 and Ki-67 expression, freshly isolated cells were fixed and permeabilized using Fixation/Permeabilization buffer kit (eBioscience) according to the manufacturer's instructions and as previously described^70^.

### Humanized mice and purification of human ILC2

For human peripheral ILC2s, peripheral blood mononuclear cells (PBMCs) were first isolated from human fresh blood by diluting the blood 1:1 in PBS, adding to SepMate-50 separation tubes (STEMCELL Technologies Inc, Vancouver, Canada) prefilled with 15-ml Lymphoprep each (Axis-Shield, Oslo, Norway), and centrifuging at 1,200*g* for 15 min. Human PBMCs were then washed in PBS and stained with antibodies against human lineage markers (CD3, CD14, CD16, CD19, CD20, CD56, CD235a, CD1a, CD123), CRTH2, CD161, CD127 and CD45. Thereafter, human ILC2s were defined as CD45+ lineage- CRTH2+ CD127+ CD161+ and purified by flow cytometry using BD FACS ARIA III (BD Biosciences, San Jose, CA) with a purity of >95%. Purified human ILC2s were cultured with rh-IL2 (20 ng ml^−1^) and rh-IL-7 (20 ng ml^−1^) for 48 h, then adoptively transferred to Rag2 GC double knockout mice (2.5 × 10^5^ cells per mouse) followed by i.n. administration of recombinant human IL-33 (0.5 μg per mouse) with or without α7nAChR agonist on days 1–3. On day 4, lung function was measured and BAL was performed and analysed.

### *In vitro* stimulation of ILC2s

Murine ILC2s were isolated from BALB/cByJ mice to >95% purity using the FACSARIA III cell sorter. Isolated murine ILC2s (5.0 × 10^4^ per ml) were stimulated with rm-IL-33 (20 ng ml^−1^), rm-IL-2 (10 ng ml^−1^) and rm-IL-7 (10 ng ml^−1^) in the presence or absence of increasing doses of α7nAChR agonist (2.5 μg ml^−1^, 10 μg ml^−1^ and 50 μg ml^−1^) for 1 or 2 days. Isolated murine ILC2s (5.0 × 10^4^ per ml) were stimulated with rm-IL-25 (10 ng ml^−1^) in the presence or absence of α7nAChR agonist (10 μg ml^−1^) for 24 h. For human ILC2s *in vitro* culture, isolated human ILC2s (1.0 × 10^5^ ml^−1^) were cultured with rh-IL-33 (20 ng ml^−1^), rh-IL-2 (10 ng ml^−1^), and rh-IL-7 (20 ng ml^−1^) in the presence of α7nAChR (10 μg ml^−1^). Levels of cytokines were measured by ELISA (eBioscience), according to the manufacturer's instructions.

### Reverse transcription-PCR

The expression of α7 and α4 genes was quantitated at the mRNA levels by quantitative real-time PCR (qPCR). Total RNA was extracted with the RNeasy Mini Kit (Qiagen, Valencia, CA), and α7nChR (forward, 5′-CTCTGACTGTCTTCATGCTGCT-3′ and reverse, 5′-ATCATGGTGCTGGCGAAG-3′) and α4nAChR (forward, 5′-CGTCCAGTACATTGCAGACC-3′ and reverse, 5′-ATGACCATGGCCACGTATTT-3′) genes were quantified at the mRNA level by quantitative real-time PCR (qPCR). Total RNA was extracted with the RNeasy Mini Kit (Qiagen, Valencia, CA), and the α7 and α4 mRNA expressions were measured using the TaqMan gene expression assay at the Applied Biosystems 7500 system (Applied Biosystems, Carlsbad, CA) in accordance with the manufacturer's protocol.

### Gene expression analysis with NanoString nCounter technology

The difference in the abundance of scripts between ILC2s purified from rm-IL33 administered mice with or without α7nAChR agonist were analysed with NanoString nCounter technology. Heat plots were generated with R statistical software.

### Statistical analysis

A student *t*-test was used for comparisons between each group. *P* values of <0.05 were considered significant. All data are expressed as the mean±s.e.m.

### Illustrations

In Figs 3, 4, 6 and 7 and thumbnail picture we used vector elements from Servier Medical Art PowerPoints under a Creative Commons License.

### Data availability

The authors declare that the data supporting the findings of this study are available within the article and its Supplementary Information files.

## Additional information

**How to cite this article:** Galle-Treger, L. *et al*. Nicotinic acetylcholine receptor agonist attenuates ILC2-Dependent airway hyperreactivity. *Nat. Commun.* **7,** 13202 doi: 10.1038/ncomms13202 (2016).

## Supplementary Material

Supplementary Information Supplementary Figures 1-3
