# Supplementary material for: Nicotinic acetylcholine receptor agonist attenuates ILC2-dependent airway hyperreactivity
Source: Nat Commun. 2016 Oct 18;7:13202. doi: 10.1038/ncomms13202 (PMC5071851; doi:10.1038/ncomms13202)
Supplement: Supplementary Information — Supplementary Figures 1-3 [file ncomms13202-s1.pdf]

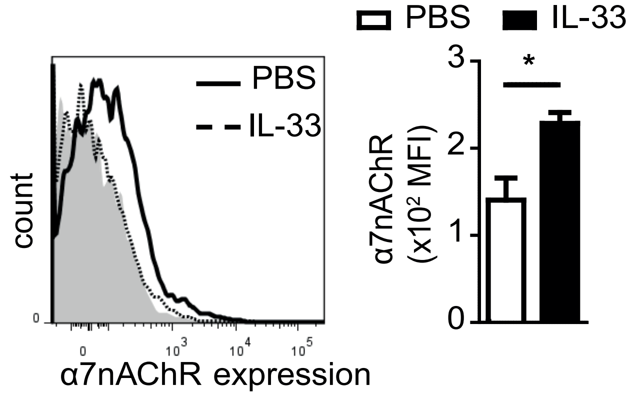

Supplementary Figure 1. Detection of α7nAChR in ILC2s by α-bungarotoxin

Histogram (left panel) and mean fluorescence intensity (right panel) of α7nAChR expression in ILC2s from BALB/cByJ mice after i.n. IL-33 (thick line) or PBS (dotted line).
The level isotype-matched stain control is shown as a gray-filled histogram (left panel).
Data are representative of at least two independent experiments and are presented as means ± SEM (n=3; Student’s t test *, $p<0.05$).

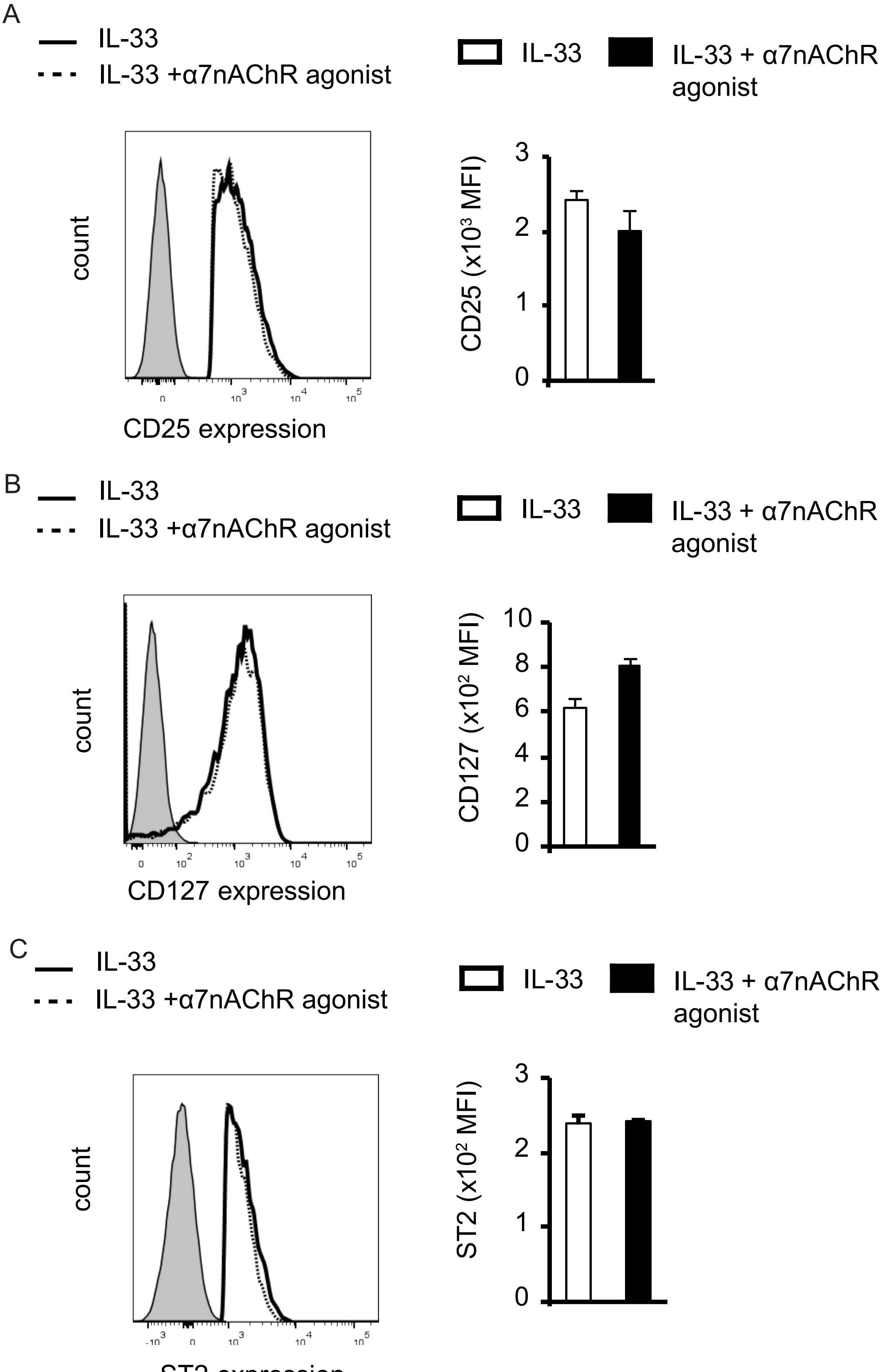

Supplementary Figure 2. Effect of α7nAChR agonist on some marker expressions by ILC2s

A cohort of BALB/cByJ mice were intranasally challenged with rm-IL-33 (0.5μg) with or without α7nAChR agonist (125μg) for three consecutive days. Histogram and MFI of CD25 (A), CD127 (B) and ST2 (C) in isolated lung ILC2s. Data are representative of at least two independent experiments and are presented as means ± SEM (n=4; Student's t test *, $p<0.05$).

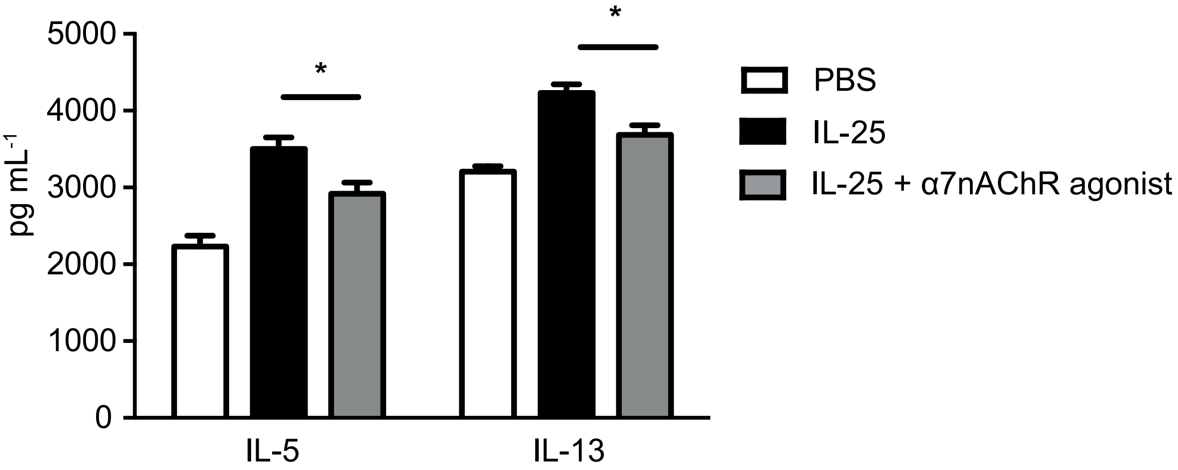

Supplementary Figure 3. α7nAChR agonist suppresses cytokine production in ILC2s

Lung ILC2s were sorted from naïve BALB/cByJ mice and cultured with rm-IL-25 (10ng mL^{-1}) in the presence or absence of α7nAChR (10μg mL^{-1}) agonist for 24 hours. The levels of IL-5 and IL-13 were measured by ELISA.
Data are representative of at least two independent experiments and are presented as means ± SEM (n=4; Student's t test *, $p<0.05$).
